# Myofibroblasts derived type V collagen promoting tissue mechanical stress and facilitating metastasis and therapy resistance of lung adenocarcinoma cells

**DOI:** 10.1038/s41419-024-06873-6

**Published:** 2024-07-10

**Authors:** Guangsheng Zhu, Yanan Wang, Yingjie Wang, Hua Huang, Boshi Li, Peijie Chen, Chen Chen, Hongbing Zhang, Yongwen Li, Hongyu Liu, Jun Chen

**Affiliations:** 1 Department of Lung Cancer Surgery, Tianjin Medical University General Hospital, Tianjin, People’s Republic of China; 2 Tianjin Key Laboratory of Lung Cancer Metastasis and Tumor Microenvironment, Tianjin Lung Cancer Institute, Tianjin Medical University General Hospital, Tianjin, People’s Republic of China

**Keywords:** Prognostic markers, Cancer microenvironment, Non-small-cell lung cancer

## Abstract

Lung cancer is a leading cause of cancer-related mortality globally, with a dismal 5-year survival rate, particularly for Lung Adenocarcinoma (LUAD). Mechanical changes within the tumor microenvironment, such as extracellular matrix (ECM) remodeling and fibroblast activity, play pivotal roles in cancer progression and metastasis. However, the specific impact of the basement membrane (BM) on the mechanical characteristics of LUAD remains unclear. This study aims to identify BM genes influencing internal mechanical stress in tumors, elucidating their effects on LUAD metastasis and therapy resistance, and exploring strategies to counteract these effects. Using Matrigel overlay and Transwell assays, we found that mechanical stress, mimicked by matrix application, augmented LUAD cell migration and invasion, correlating with ECM alterations and activation of the epithelial-mesenchymal transition (EMT) pathway. Employing machine learning, we developed the SVM_Score model based on relevant BM genes, which accurately predicted LUAD patient prognosis and EMT propensity across multiple datasets. Lower SVM_Scores were associated with worse survival outcomes, elevated cancer-related pathways, increased Tumor Mutation Burden, and higher internal mechanical stress in LUAD tissues. Notably, the SVM_Score was closely linked to COL5A1 expression in myofibroblasts, a key marker of mechanical stress. High COL5A1 expression from myofibroblasts promoted tumor invasiveness and EMT pathway activation in LUAD cells. Additionally, treatment with Sorafenib, which targets COL5A1 secretion, attenuated the tumor-promoting effects of myofibroblast-derived COL5A1, inhibiting LUAD cell proliferation, migration, and enhancing chemosensitivity. In conclusion, this study elucidates the complex interplay between mechanical stress, ECM alterations, and LUAD progression. The SVM_Score emerges as a robust prognostic tool reflecting tumor mechanical characteristics, while Sorafenib intervention targeting COL5A1 secretion presents a promising therapeutic strategy to mitigate LUAD aggressiveness. These findings deepen our understanding of the biomechanical aspects of LUAD and offer insights for future research and clinical applications.

## Introduction

Lung cancer is the most common cancer, accounting for 11.6% of all newly diagnosed malignancies. It is dominated by non-small cell lung cancer (NSCLC), which accounts for approximately 85% of lung cancer cases [1, 2]. Lung cancer is the leading cause of male and female cancer deaths in over 90 countries, partly due to its high mortality rate [1]. Lung adenocarcinoma (LUAD), which is one of the significant NSCLC histology types, has increased in prevalence compared to other lung cancer subtypes [3], and the 5-year survival rate is only approximately 5% for LUAD.

The changes in the mechanical environment produce deformation and movement effects on tissue cells and lead to complex physiological and pathological physiological changes [4], including lung cancer. Emerging evidence suggests that mechanical alterations and an abnormal microenvironment may cooperatively drive cancer cell aggression and treatment resistance [5]. The tumor microenvironment plays an important role during tumor progression and runs throughout [6]. The pathological changes in the tumor microenvironment, including inflammatory exudation, cell infiltration and contraction, and extracellular matrix remodeling, promote tumor progression while also altering the mechanical characteristics of the tumor [7, 8]. Among them, the contraction of fibroblasts and the hardening of the extracellular matrix are essential factors that alter the mechanical characteristics of the tumor microenvironment [7].

The extracellular matrix (ECM) is involved in a variety of activities, such as regulating cell growth, migration, and differentiation, and also a major tumor-stroma component [9, 10]. Meanwhile, the quantity and cross-linking status of ECM components determine tissue stiffness. The physical reorganization of collagen causes the ECM to gradually while the internal stress of the tumor increases. Eventually, due to the hardening of the stroma and the change in stress [11, 12], the tumor cell polarity and the intercellular adhesion changes, leading to tumor cell migration along the hardened collagen fibers toward the stroma and facilitating distant metastasis of the tumor [11, 13].

The basement membrane (BM), a special ECM arranged on the bottom side of epithelial and endothelial tissues, stabilizes the tissue structure and is essential for biological behaviors such as cell proliferation, differentiation, angiogenesis, and tissue repair [14, 15]. In addition, aberrant BM gene expression is thought to be associated with diseases such as tumors. Tumor cells are thought to have a greater ability to migrate and invade when they break through the BM [16]. Epithelial tissue formation during animal development depends on mechanical force generation and the sensitivity of cells to mechanical stress [17], and the BM is inevitably subjected to mechanical stress as an extracellular matrix. During malignant tumor progression, tumor cells induce the generation of intracellular contractile forces through adhesion to the pericellular matrix, altering the mechanical stresses in the TME, and an increase in tumor-associated collagen prompts tumor cell adaptation to the mechanical changes induced by the mechanical stresses on the BM [18]. Mechanical stresses within the tumor stiffen the BM increasing the risk of invasive cancer development. Tumor stromal sitffening may further promote the migration and invasiveness of malignant cells [19].

In contrast, sorafenib, as an oral multikinase inhibitor, blocks TGF-β signaling, decreasing the expression of collagen and pro-fibrotic genes, resulting in a reduction of tumor-stroma stiffness and a weakening of inter-tumor stress. This inhibits the fibrosis process, reduces the proliferation of fibroblasts and the production of Epithelial-Mesenchymal Transition (EMT), attenuates the accumulation effect of ECM, and inhibits tumor migration and invasion [20, 21]. Therefore, sorafenib has the potential to reduce the internal mechanical stress of tumors.

Understanding how changes in the tumor microenvironment’s mechanical characteristics affect tumors’ biological characteristics and clarifying the signaling pathways related to cellular mechanical mechanics is an urgent page to be completed. Simultaneously, BM genes are closely associated with modulating mechanical stress within tumors. Therefore, this study will commence from the perspective of BM genes to investigate the mechanisms by which internal mechanical stress influences tumors.

## Methods

### Cell culture and transfection

H1975 and A549 LUAD cell lines were obtained from the ATCC and cultured in RPMI 1640 and DMEM medium, respectively, supplemented with 10% FBS and maintained at 37 °C in a 5% CO_2_ humidified atmosphere. After cell seeding, a layer of extracellular 17 mg/ml Matrigel was overlaid to impart mechanical stress [22]. To perform the transfection, siRNA explicitly targeting COL5A1 or a scrambled control siRNA (Ribobio,) was transfected using Lipofectamine 2000 (Invitrogen, USA) following the manufacturer’s instructions. The siRNAs targeting COL5A1 were sense: 5′-GGGAUUCCUUCAAGGUUUATT-3′, antisense: 5′-UAAACCUUGAAGGAAUCCCTT-3′.

### Western blot

Protein samples were extracted and quantified, and agarose gel electrophoresis was performed using 10% separating gels. Proteins were transferred onto PVDF membranes (Millipore, Billerica, MA, USA) using a semidry transfer system. The membranes were then blocked with 5% skim milk at room temperature for 2 h. The primary antibodies, anti-beta-catenin (Affinity, BF8016, 1:1000), anti-Snail (CST, 3879S, 1:1000), anti-E-cadherin (BD, 810182S, 1:1000), anti-Fibronectin (CST, 26836, 1:1000), anti-Vimentin (Pronteintech, 10366-1-AP, 1:1000), anti-GAPDH(Pronteintech, 60004-1-lg, 1:1000) were incubated with membranes overnight at 4 °C. Subsequently, the secondary antibody was added and(1:5000 dilution; Thermo Fisher Scientific, Inc.) incubated for 1 h at room temperature. The bands were visualized using the Pierce ECL substrate (Thermo Fisher Scientific, Inc.).

### Immunofluorescence and immunohistochemistry (IHC)

LUAD cells were seeded on 12-well plates with coverslips, fixed in 4% paraformaldehyde, permeabilized with 0.5% Triton X-100, blocked with 1% BSA, and incubated with the primary antibodies, such as anti-panCK (Abcam, ab7753, 1:1000), anti-COL5A1(Immunoway, YT1030, 1:200), anti-Vimentin (Pronteintech, 10366-1-AP, 1:200), anti-alpha-SMA (Immunoway, Y5053, 1:200)) overnight at 4 °C. After washing, cells were incubated with the fluorescent secondary antibody, and coverslips were mounted using a fluorescence quenching mounting medium with DAPI. Imaging was performed using a fluorescent microscope. IHC staining of paraffin-embedded tissues with primary antibodies (anti-COL5A1(Immunoway, YT1030, 1:200), anti-Vimentin (Pronteintech, 10366-1-AP, 1:200), anti-alpha-SMA (Immunoway, Y5053, 1:200), and anti-Ki67 (Abcam, ab1667, 1:200) were performed and scored according to standard procedures. Two independent pathologists determined the staining score.

### Transwell assay

Cell migration and invasion were evaluated using transwell chambers. For the migration assay, cells were seeded into the upper chamber with a serum-free medium, whereas the lower chamber was filled with a medium containing 10% fetal bovine serum. For the Matrigel group, 17 mg/ml Matrigel was added above to the cells to provide additional mechanical stress after planting the cells. After incubation for 24 h, the cells that migrated to the lower chamber were fixed with 4% paraformaldehyde and stained with crystal violet. For the invasion assay, transwell chambers were coated with Matrigel before cell seeding.

### Scratch wound-healing assay

The cells were seeded into 6-well plates and cultured to confluence. A scratch wound was created using a sterile 200 μL pipette tip, and the cells were washed with phosphate-buffered saline to remove the debris. Then, the cells were incubated in a serum-free medium, and wound closure was monitored at various time points using an inverted microscope. For the Matrigel group, after planting the cells, 17 mg/ml Matrigel was added to the above cells to provide additional mechanical stress.

### Data sources of the bioinformatics analyses and lung cancer tissue collection

Seven data sources were collected in our study, including the Cancer Genome Map (TCGA) database (59 normal and 515 lung adenocarcinoma samples), GSE3141 (58 lung adenocarcinoma samples), GSE26939 (86 lung adenocarcinoma samples), GSE30219 (83 lung adenocarcinoma samples), GSE31210 (226 lung adenocarcinoma samples), GSE50081 (127 lung adenocarcinoma samples), GSE131907 (11 lung adenocarcinoma samples, 25,369 cells) and GSE72094 (398 lung adenocarcinoma samples) and downloaded the transcriptional profiles of bulk RNA sequencing and single-cell RNA sequencing for all tumor samples, as well as relevant clinical information. Two hundred and twenty-two BM-related genes were derived from a recently published study [23]. To facilitate the smooth progress of the research, we obtained 35 BM-related genes for subsequent research by intersecting the seven studies above and the BM-related genes. Transcript sequencing for 33 LUAD patients was obtained from the Department of Lung Cancer Surgery of Tianjin Medical University General Hospital (TMU), and tumor specimens from ten of those patients were performed with immunohistochemistry (IHC) and polychromatic immunofluorescence. The informed consent was obtained from each patient.

### Selection and understanding of candidate BM genes

First, differential expression analysis was performed on normal and lung cancer samples of TCGA-LUAD using the R package “edgR” [20] and displayed through heat maps and volcanic maps. Somatic mutations with differentially expressed BM genes were visualized using the R package “maftools” [24]. Then, to further screen for BM core genes, a univariate Cox analysis of variance was applied to determine OS-related genes.

### Signature generated from machine learning-based integrative approaches

We have integrated ten machine learning algorithms and 101 algorithm combinations. The comprehensive algorithms include Random Survival Forest, Elastic Network (Enet), Lasso, Ridge, Stepwise Cox, CoxBoost, Cox Partial Least Squares Regression (plsRcox), Supervised Principal Component, Generalized Enhanced Resignation Modeling (GBM), and Survival Support Vector Machine (Survival SVM). The feature generation program is as follows: First, univariate Cox regression in the TCGA-LUAD queue was used to determine prognosis-related BM genes. Then, in the TCGA-LUAD queue, 101 algorithm combinations were performed on relevant BM genes to establish a prediction model based on the left one cross-validation (LOOCV) framework. Subsequently, all models (GSE3141, GSE26939, GSE30219, GSE31210, GSE50081, and GSE72094) were detected in six validation datasets. Finally, the Harrell consistency index (C-index) is calculated on all validation datasets for each model, and the model with the highest average C-index was considered optimal.

### Bioinformatics and statistical methods

Immune microenvironment scoring [25] and immune cell infiltration analysis were performed using the GSVA package [26]. Drug sensitivity analysis was carried out using the pRRophytic package [27]. Single-cell analysis was completed by the Seurat package [28]. Cell Chat analysis was completed by the CellChat package [29]. An independent sample t-test was used to compare two sample groups. The Wilcoxon test compared multiple groups of samples, and the log-rank test was used to compare two or more survival curves. Essential scripts for implementing machine learning-based integrative procedures in multiple independent datasets are available on the GitHub website (https://github.com/Zaoqu-Liu/IRLS). Essential single-cell analysis scripts are available on the GitHub website (https://github.com/Mcdull8/METArisk). Statistical significance was defined as a *p*-value ≤ 0.05. **** denotes a *p*-value ≤ 0.0001, *** denotes a *p*-value ≤ 0.001, ** denotes a *p*-value ≤ 0.01, and * denotes a *p*-value ≤ 0.05.

### Atomic force microscopy(AFM)

The Park NX10 atomic force microscope (manufactured by Park Systems, South Korea) was utilized for AFM measurements on each slice. Based on TL-CONT-NANOSENSORS, cantilever probes with ten μm silicon dioxide spheres were used for detection. Before each experiment, a noncontact calibration method was used to calibrate each probe’s sensitivity and spring constant. All measurements were conducted in force spectroscopy mode, and force-indentation curves were generated from an average of 20 points per sample. All force measurements’ approach and retraction speeds were set at 0.2 μm/s, with a set point force of 2.5 nN and a retraction distance of 0.5 μm. XEI data processing software was used for data analysis. The Young’s modulus was calculated from AFM force curves using the Hertz-Sneddon model to assess tissue stiffness. Young’s modulus data were plotted in GraphPad software, and intergroup differences were tested using t-tests.

### Cell proliferation assay

Cell proliferation was evaluated utilizing the Cell Counting Kit-8 (CCK8) assay. Cells were plated in 96-well plates and cultured for 24, 48, and 72 h. At each time point, 10 μL of CCK8 solution was introduced to individual wells and incubated for 2 h. Subsequently, the optical density was quantified at 450 nm using a microplate reader.

### Establishment and culture of LUAD tumor organoids

Tumor specimens were dissected into small pieces and incubated in a tissue digestion medium containing Advanced DMEM/F-12 (Gibco), 200 mM GlutaMAX (Gibco), 1 M HEPES (Gibco), 1× Primocin (InvivoGen), 1 mg/ml collagenase IV (Sigma-Aldrich), 100 μg/ml DNase I (AppliChem), 1× B27 (Gibco), 1 mM N-acetyl cysteine (Sigma-Aldrich), and 10 μM Y-27632 (Selleckchem). The cells were resuspended in growth factor-reduced Matrigel (Corning) and seeded in 12-well plates as 50 μl droplets. After 15 min at 37 °C, 1 mL of culture medium containing Advanced DMEM/F-12, 200 mM GlutaMAX, 1 M HEPES, 1× B27 supplement, 1 mM N-acetyl cysteine, 10% RSPO1-conditioned medium, 100 ng/ml FGF-10 (PeproTech), 100 ng/ml Noggin (PeproTech), 500 nM A83-01 (Tocris), and 1× Primocin was added. Organoids were passaged approximately every seven days by dissociation at 37 °C using TrypLE (Gibco).

### Isolation and culture of myofibroblasts

Primary myofibroblasts were isolated from tumor specimens using a growth-based method [30]. During the organ separation process, tissue fragments were generated and co-cultured with fibroblast culture medium containing 10% fetal bovine serum (Gibco), 200 mM GlutaMAX (Gibco), and penicillin/streptomycin (Gibco) in RPMI medium. The culturing cancer-associated fibroblasts (CAFs) success rate was approximately 90%. CAFs were passaged approximately every 8–9 days, and their identity was confirmed through morphological assessment and immunofluorescence staining for α-SMA. CAFs were used for experiments within 4–7 passages after isolation.

### Co-culture of tumor organoids and Myofibroblasts

The digestion of organoid into single cells or small aggregates (termed organoid-forming units) was performed using TrypLE Express (Gibco), supplemented with 100 μg/ml of DNase I (from AppliChem) and 10 μM of Y-27632. A 1:1 mixture of organoid-forming units and fibroblasts was prepared. Depending on the growth rate of each organoid system, approximately 2000–3000 organoid-forming units were utilized for every 10 μl of matrix. Cells were seeded into a co-culture matrix in droplet form, consisting of a gelatin solution and 3 mg/ml of collagen I (from Corning). The co-culture system was maintained in a medium containing Advanced DMEM/F12, 200 mM GlutaMAX, 1 M HEPES, 1× B27, 100 ng/ml of FGF-10, 50 ng/ml of EGF (obtained from PeproTech), and 5% RSPO1-conditioned medium [31].

## Results

### Mechanical stress upregulates tumor EMT pathway and promotes tumor migration and invasion ability

**Fig. 1:**
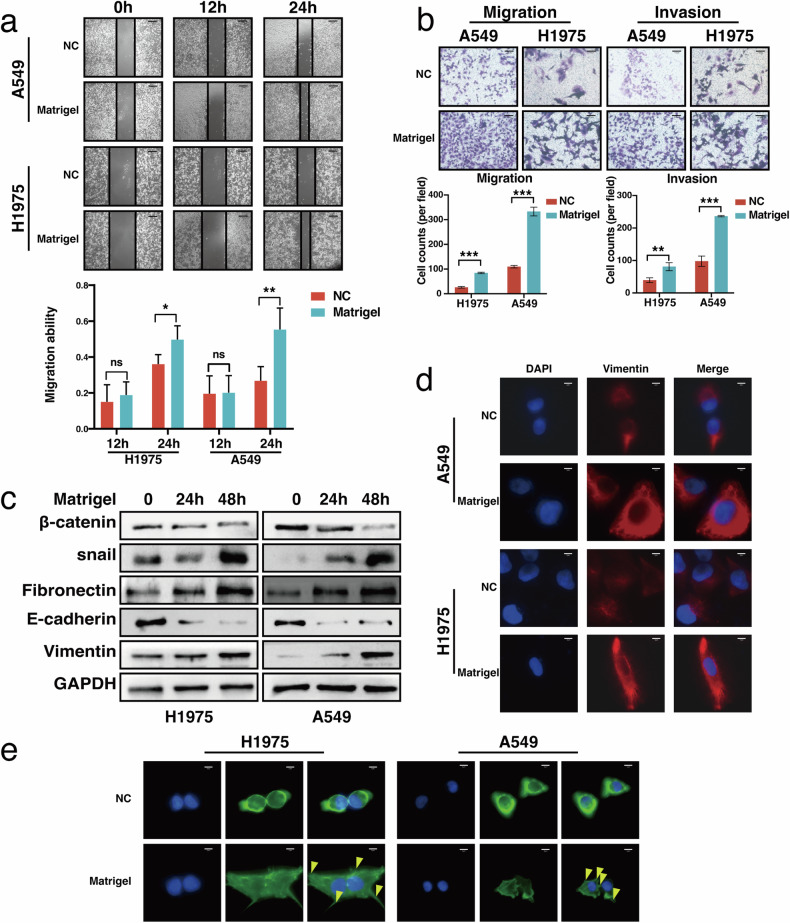
Mechanical stress enhances tumor metastasis and upregulates the EMT pathway. The migration and invasive ability of LUAD cells were tested by scratch wound-healing assay (**a**) and transwell assays (**b**). **c** EMT marker expression in response to Matrigel addition was measured with Western blot. **d** Vimentin expression changes were visualized with an immunofluorescence assay. **e** Cell morphology and b-actin alterations were visualized with Phalloidin Staining (Yellow arrow: tentacles).

We added Matrigel to the scratched LUAD cells seeded on 6-well plates to investigate the influence of mechanical stress to the migration and invasive ability of LUAD cells. The A549 and H1975 cells had enhanced migration ability after adding matrix adhesive and were significantly superior in 24-hour migration ability compared to their counterparts maintained without Matrigel (Fig. 1a). When testing lung adenocarcinoma migration ability through the transwell experiments (Fig. 1b), we also found that the tumor cells with added Matrigel above achieved stronger migration ability and had statistical significance. Similarly, when testing the invasive ability of lung adenocarcinoma cells through the Transwell experiment, we also found that the two types of tumor cells with added matrix glue achieved significantly stronger invasive ability than their counterparts maintained without Matrigel. (Fig. 1b).

The Western blot experiment showed that the EMT pathway of tumor cells was significantly upregulated when adding the matrix gel, manifested as the upregulation of Vimentin and Snail and downregulation of beta-Catenin and E-cadherin (Fig. 1c). Additionally, the immunofluorescence experiments showed that the tumor cells with matrix glue added significantly upregulated Vimentin (Fig. 1d). The Phalloidin experiment demonstrated that the tumor cell cultured with matrix gel changed morphology significantly and were no longer round and continuous, with a significant increase in tentacles (Fig. 1e).

### SVM_Score generated based on machine learning effectively predicts the prognosis of lung adenocarcinoma patients

A heat map between normal and lung adenocarcinoma tissues of TCGA-LUAD is shown in Supplementary Fig. 1a. The upste plot then shows 35 BM genes in all seven datasets we used (Supplementary Fig. 1b). The volcanic map displays the differential genes between normal and lung adenocarcinoma tissues of TCGA-LUAD (Supplementary Fig. 1c). At the same time, we conducted unit correlation analysis on these 35 BM genes and selected 14 BM genes related to the overall survival of lung adenocarcinoma for subsequent modeling by intersecting with differential gene results (Supplementary Fig. 1d). The oncoplt showed the mutations status of these 14 genes in lung adenocarcinoma, with the FBN2 gene has the highest mutation rate of 16%. (Supplementary Fig. 1e).

**Fig. 2:**
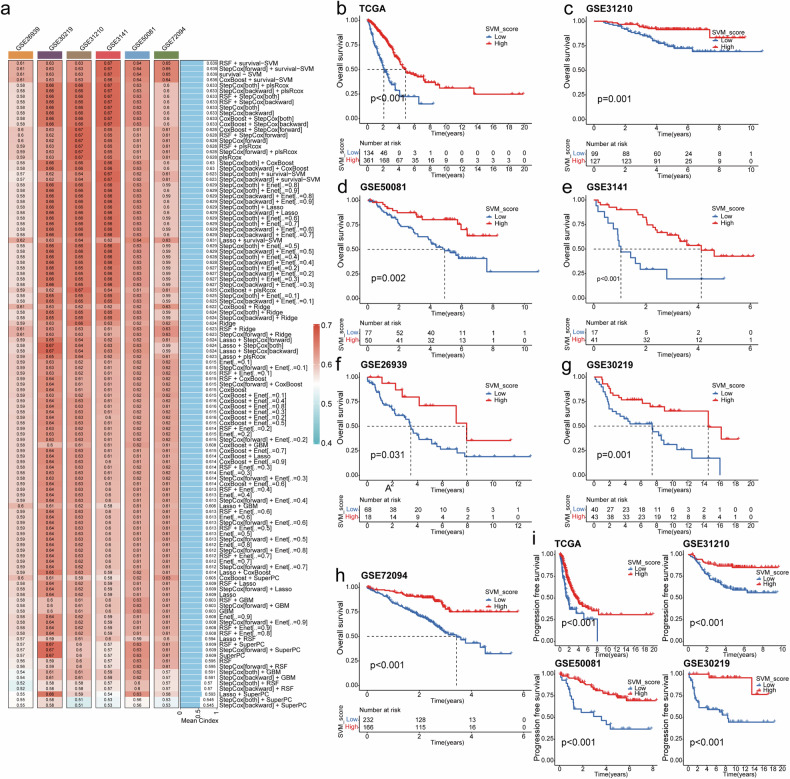
Construct a prognosis prediction model based on BM genes using machine learning algorithms and validate with multiple datasets. **a** Build and screen a prognosis prediction model with machine learning algorithms based on BM genes. **b**–**i** Kaplan–Meier survival analysis for overall survival based on SVM_Scores in various datasets, including TCGA-LUAD (**b**), GSE31210 (**c**), GSE50081 (**d**), GSE3141 (**e**), GSE26939 (**f**), GSE30219 (**g**), and GSE72094 (**h**). **i** Progression-free survival analysis based on SVM_Scores in TCGA-LUAD, GSE31210, GSE50081, and GSE30219.

Our machine learning analysis results indicate that SVM_score, constructed using a Support Vector Machine (SVM), has the highest Concordance Index(C-index), as shown in Fig. 2a. C-index is a statistical measure used to assess a model’s discriminatory power or predictive accuracy, particularly in the context of survival analysis or time-to-event data. A higher C-index indicates a model with good discrimination. Therefore, we selected SVM_score for further analysis. Survival analysis revealed that in TCGA-LUAD (Fig. 2b), GSE31210 (Fig. 2c), GSE50081 (Fig. 2d), GSE3141 (Fig. 2e), GSE26939 (Fig. 2f), GSE30219 (Fig. 2g), and GSE72094 datasets (Fig. 2h), lower SVM_score values corresponded with a shorter overall survival. Additionally, in TCGA-LUAD, GSE31210, GSE50081, and GSE30219 datasets, lower SVM_score values were associated with shorter progression-free survival (Fig. 2i).

**Fig. 3:**
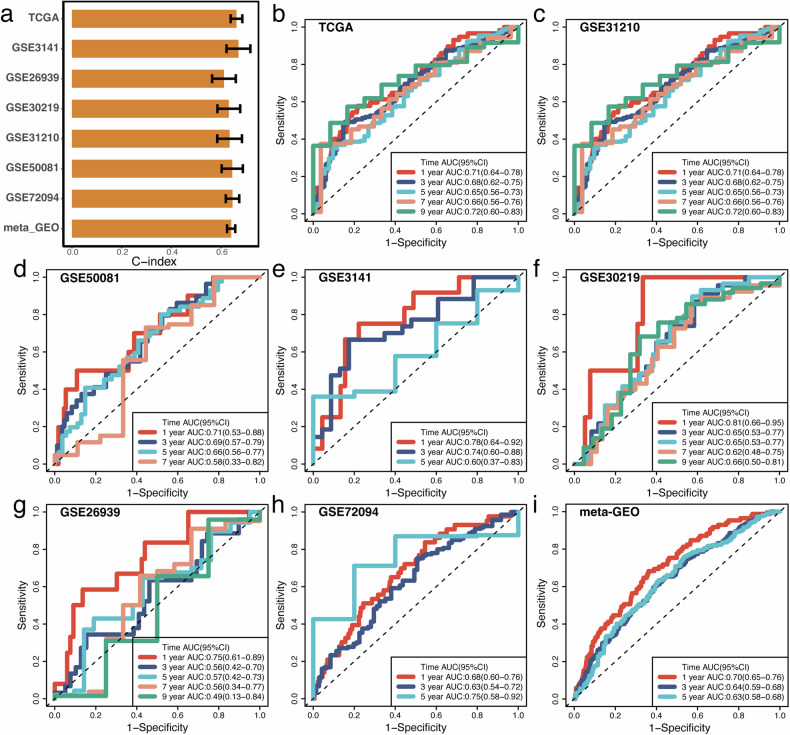
Predictive performance of SVM_Score in patient outcome prediction. (**a**) C-index Values: Comparison of C-index values for SVM_Score and other prognostic indicators, evaluating their effectiveness in predicting patient outcomes. **b**–**i** AUC Analysis with SVM_Score in the various datasets, including TCGA-LUAD (**b**), GSE31210 (**c**), GSE50081 (**d**), GSE3141 (**e**), GSE30219 (**f**), GSE26939 (**g**), GSE72094 (**h**) and meta-GEO (**i**).

SVM_score demonstrated robust prognostic prediction capabilities in TCGA-LUAD, GSE31210, GSE50081, GSE3141, GSE26939, GSE30219, and GSE72094, with C-index values exceeding 0.6 across all seven datasets (Fig. 3a). Furthermore, the area under the curve (AUC) values for one-year overall survival, determined by ROC analysis, exceeded 0.7 in TCGA-LUAD (Fig. 3b), GSE31210 (Fig. 3c), GSE50081 (Fig. 3d), GSE3141 (Fig. 3e), GSE26939 (Fig. 3f), and GSE30219 (Fig. 3g), with GSE30219(Fig. 3h) even surpassing 0.8. The one-year overall survival AUC reached 0.7 when combining all GEO datasets (Fig. 3i).

The c-index of SVM_score, compared to other commonly used clinical factors in the TCGA-LUAD dataset, is slightly lower than the Stage factor but higher than all other factors (Supplementary Fig. 2a). In the meta-GEO dataset, SVM_score outperforms all other factors, including Stage (Supplementary Fig. 2b). Similarly, in the GSE31210 dataset, SVM_score surpasses all other factors except for Stage (Supplementary Fig. 2c). In the GSE50081 dataset, SVM_score outperforms all other factors (Supplementary Fig. 2d). In the GSE26939 dataset, SVM_score exceeds all other indicators except age (Supplementary Fig. 2e). Finally, in the GSE30219 (Supplementary Fig. 2f) and GSE70294 (Supplementary Fig. 2g) datasets, SVM_score outperforms all other factors.

### SVM_Score is associated with tumor proliferation and EMT propensity in TMU LUAD patients

**Fig. 4:**
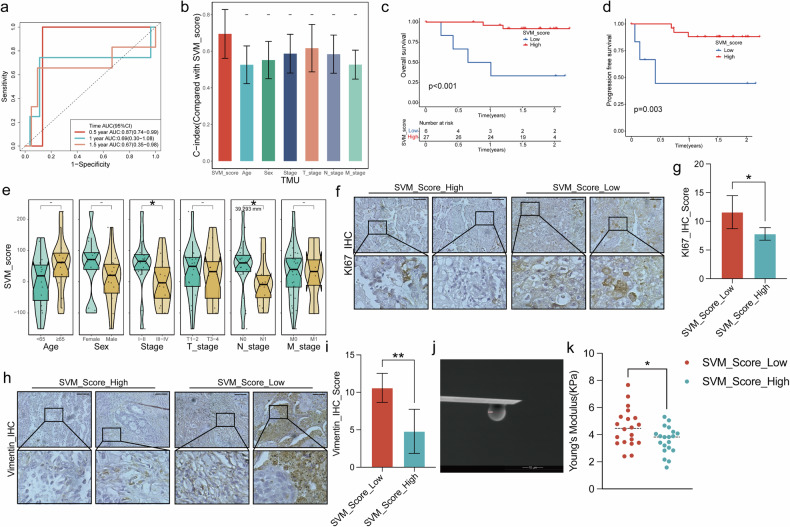
SVM_Score prognostic prediction capabilities in Tianjin Medical University (TMU) LUAD patients. **a** Using SVM_Score in the performed ROC analysis on overall survival rate for TMU LUAD patients. **b** Comparison of C-index values and SVM_Score and other clinical factors with TMU LUAD patients. Survival analysis illustrates lower SVM_Score in the TMU LUAD patients associated with poor overall survival (**c**) and progression-free survival (**d**). **e** Check SVM_Scores in TMU LUAD patients stratified by clinical factors. Representative images of immunohistochemical results in ten TMU LUAD patients with low/high SVM_Scores staining with Ki67 (**f**, **g**) and Vimentin (**h**, **i**). Patients with lower SVM_Scores had significantly higher Ki67 (**g**) and Vimentin (**i**). **j** Electron microscopy scan image of the probe of atomic force microscope. **k** Comparison of tumor tissue Young’s modulus value between patients with high and low SVM_Scores.

We further investigate the SVM_Score in the LUAD from our institute. In the 33 cases of TMU LUAD patients, the one-year overall survival AUC value for SVM_score reached 0.87 (Fig. 4a). Although the C-index of SVM_score may not have achieved statistical significance compared to other clinical factors, possibly due to the limited sample size, it demonstrated a stronger trend than all other clinical factors (Fig. 4b). Survival analysis results revealed that a lower SVM_score was associated with a worse prognosis, both in terms of overall survival (Fig. 4c) and progression-free survival (Fig. 4d). We also assessed whether there were differences in SVM_score among various clinical factors, and the results indicated an association between SVM_score and the lymph node metastasis status of lung adenocarcinoma (Fig. 4e).

Immunohistochemistry results further demonstrated that patients with lower SVM_score had more tumor cell expression Ki67, indicating a higher degree of malignancy consistent and statistically significant in the ten patients (Fig. 4f–g). Additionally, tumor tissues from patients with low SVM_score showed increased expression of Vimentin, signifying higher EMT activation pathway and an increased metastasis propensity(Fig. 4h). Again, this trend was consistent and statistically significant in all the patients (Fig. 4i).

Atomic force microscopy was used to detect mechanical stress within the tumor tissues. Tumor tissues from patients in the lower SVM_score group had higher internal mechanical stress (Fig. 4j, k).

### The lower SVM_score is associated with lower immunogenicity

**Fig. 5:**
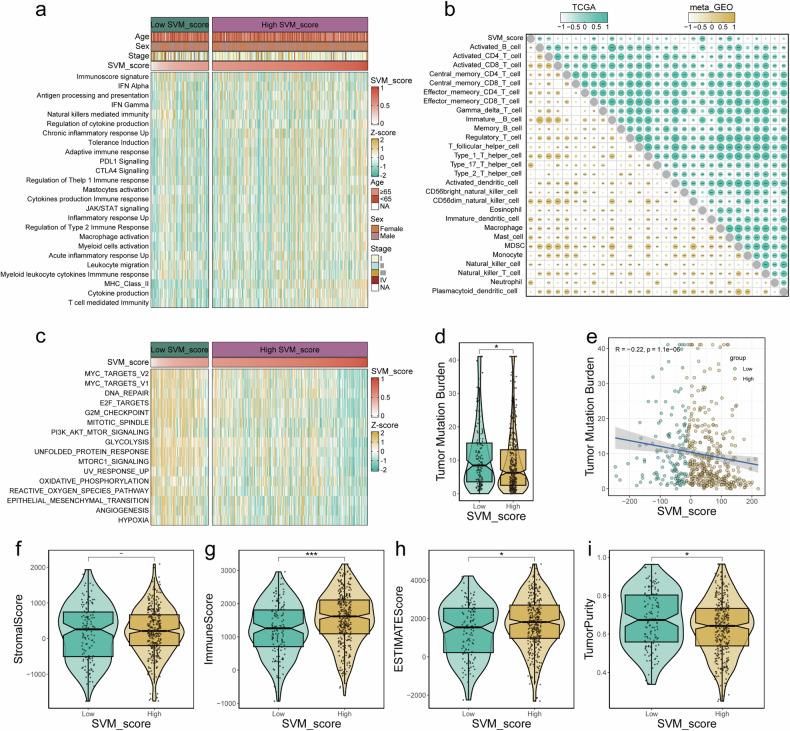
Investigation of the relationship of SVM_Score with immunogenicity and tumor microenvironment. **a** Heatmap displays the correlation between SVM_Score and immunogenicity in TCGA-LUAD. **b** Correlation analysis between SVM_Score and immune cell types in TCGA and meta-GEO datasets. **c** GSVA scores for HALLMARK cancer-related pathways, revealing differences in cancer pathway activity. **d** Analysis of Tumor Mutation Burden (TMB) in relation to SVM_Score. **e** Negative correlation between SVM_Score and TMB. The differences between the high and low SVM_Score groups in terms of stromal score (**f**), Immune score (**g**), ESTIMATE score (**h**), and Tumor purity (**i**).

We employed GSVA to calculate scores for 25 immune-related pathways (Supplementary Appendix 1) in TCGA-LUAD and displayed a heatmap illustrating lower SVM_Scores in tumor tissues associated with reduced immunogenicity (Fig. 5a). For patients with low SVM_Scores, the degree of activation of immune-related pathways is inversely correlated, indicating a lower level of immune pathway activation. We conducted separate analyses of the correlation between SVM_Scores and 27 immune cell types in TCGA and meta-GEO datasets. The results revealed statistically significant correlations between SVM scores and Activated B cell, effector memory CD8+ T cell, immature B cell, CD56dim natural killer cell, immature dendritic cell, macrophage, mast cell, and MDSC in both datasets (Fig. 5b).

Using GSVA, we calculated scores for 50 HALLMARK cancer-related pathways (Supplementary Appendix 2). Our findings demonstrated that patients with lower SVM scores exhibited increased activity in cancer pathways, including the EMT pathway (Fig. 5c). Furthermore, patients with lower SVM scores had higher Tumor Mutation Burden (TMB) (Fig. 5d), and SVM scores exhibited a negative correlation with TMB (Fig. 5e).

We employed ESTIMATE to calculate tumor stromal, immune, estimate, and tumor purity scores. We observed that SVM_Scores were independent of stromal scores (Fig. 5f). Patients with higher SVM_Scores exhibited higher immune scores (Fig. 5g) and estimate scores (Fig. 5h), as well as lower tumor purity (Fig. 5i).

### The expression of COL5A1 of myofibroblasts influences the SVM_Score, while myofibroblasts are intricately associated with the microenvironment

**Fig. 6:**
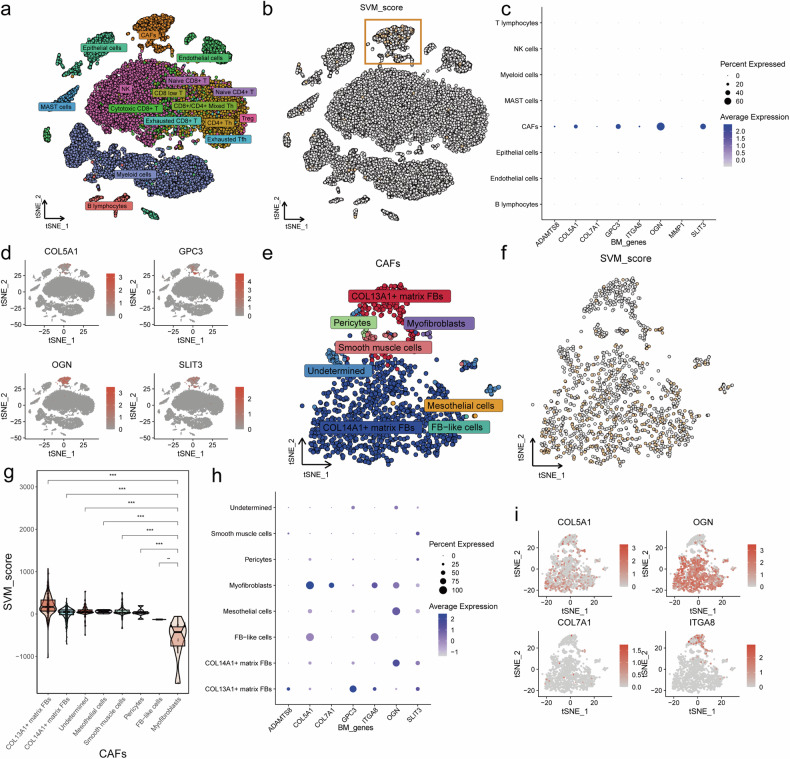
The COL5A1 of myofibroblasts impact the SVM_Score. **a**, **b** t-SNE dimensionality reduction plot and SVM_Score distribution highlighting cell clustering in different cell types. **c** Gene expression profile of SVM_Score-associated genes within various cells. **d** Expression distribution of top genes (COL5A1, GPC3, OGN, and SLT3) across the single-cell dimensionality reduction plot **e**, **f** Isolation and Distribution of SVM_Score for CAFs. **g** Comparison of SVM_Score values in myofibroblasts with other CAF subtypes. **h** Gene expression profile of SVM_Score-associated genes within CAFs. **i** Expression distribution of top genes (COL5A1, COL7A1, ITGA8, and OGN) across the CAFs single-cell dimensionality reduction plot.

To further investigate SVM_Scores among different cell populations in the LUAD patients, we applied the GSE131907 dataset, which contains single-cell sequencing data from 11 LUAD samples, including 25,369 cells. The t-SNE dimensionality reduction plot displays the types of cells in single-cell sequencing data. (Fig. 6a). We computed an SVM_Score for each cell population and visualized it on the t-SNE plot (Fig. 6b). The results showed that the SVM_Score in cancer-associated fibroblasts (CAFs) significantly differed from other cell types (Fig. 6a, b). The genes contributing to the SVM_Score were also predominantly expressed in CAFs (Fig. 6c). We presented the expression distribution of the top four genes of SVM_Score, COL5A1, GPC3, OGN, and SLT3, on the overall single-cell dimensionality reduction plot, and they were primarily localized in CAFs (Fig. 6d). Therefore, we isolated CAFs and performed a separate t-SNE dimensionality reduction (Fig. 6e). When visualizing the distribution of SVM_Scores, the results indicated that all myofibroblasts have lower SVM_Scores (Fig. 6f). Moreover, myofibroblasts exhibited significantly lower SVM_Scores than all other CAFs, except for FB-like cells, which could not be statistically analyzed due to their limited numbers (Fig. 6g). Among the genes contributing to SVM_Scores, COL5A1 showed the highest expression in myofibroblasts (Fig. 6h). The expression distribution of COL5A1 in CAFs closely resembled the SVM_Score pattern (Fig. 6i). In both the TCGA-LUAD cohort (Supplementary Fig. 3a) and the TMU patients (Supplementary Fig. 3b), COL5A1 is highly expressed in tumor tissues. The findings above regarding COL5A1 suggest that COL5A1 may serve as a pivotal biomarker linking SVM_Scores and mechanical stress.

Following that, we analyzed cellular interactions in the tumor microenvironment, which revealed that myofibroblasts were the most interactive cell type with other cells (Supplementary Fig. 4a). Furthermore, myofibroblasts exhibited the highest interaction intensity with other cells (Supplementary Fig. 4b). We also identified that myofibroblasts primarily interact by secreting Macrophage Migration Inhibitory Factor (MIF) and binding to CD74 on the cell surface of other cells, along with CXCR4 or CD44 (Supplementary Fig. 4c).

Notably, myofibroblasts predominantly interacted with other cells through the MIF signaling pathway (Supplementary Fig. 4d) and the MK signaling pathway (Supplementary Fig. 4e). Moving forward, our focus will be directed toward myofibroblasts and COL5A1.

### COL5A1 from myofibroblasts increases tumor invasiveness and upregulates the EMT pathway of tumor cells

**Fig. 7:**
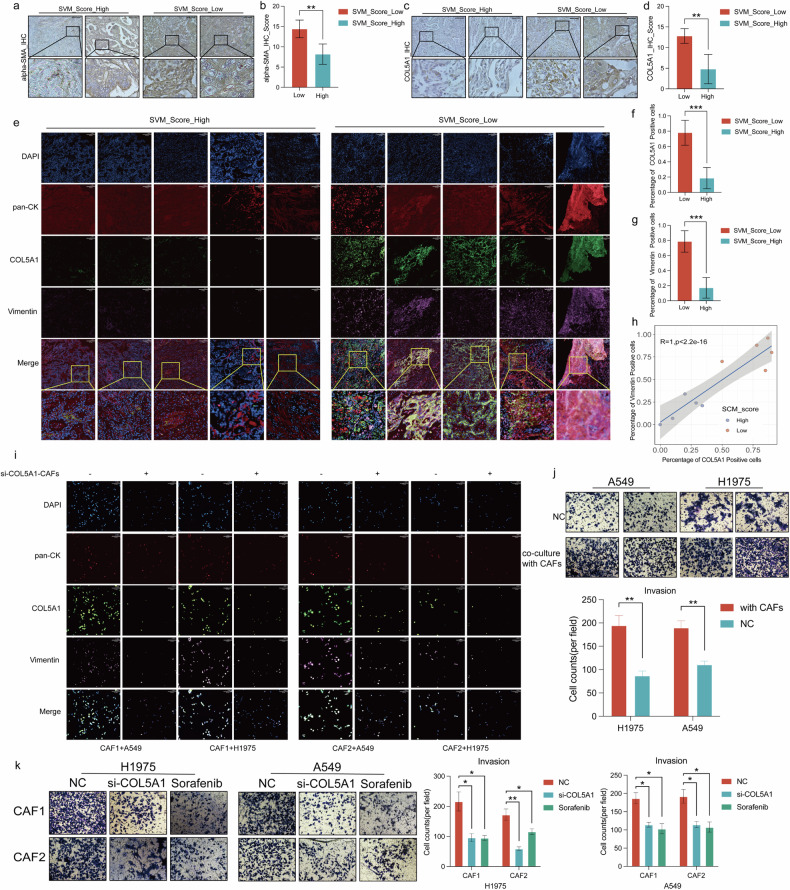
COL5A1 from myofibroblasts enhances the EMT pathway and tumor invasion ability. (**a**–**d**) The immunohistochemical results showed that in the tumor tissue of the low SVM_Score group, the expression of alpha-SMA (a, b)was statistically higher than the high SVM_Score; and the expression of COL5A1was also statistically higher (**c**, **d**). **e**–**g** Multicolor immunofluorescence analysis provides insight into elevated COL5A1 and Vimentin expression in tissues from the lower SVM_Score group (**e**). This trend is consistently observed in all ten patients and holds statistical significance (**f**, **g**). **h** Pearson correlation analysis showed a strong correlation between the number of COL5A1-positive and vimentin-positive cells. **i** Co-culturing si-COL5A1 downregulated CAF cell lines with two kinds of lung adenocarcinoma cells revealed changes in surface markers COL5A1 and Vimentin expression. **j** The Transwell experiment detected changes in the invasive ability of lung adenocarcinoma cells co-cultured with CAFs. **k** The Transwell experiment detects the invasive ability of lung adenocarcinoma cells co-cultured with CAFs, especially after knocking down COL5A1 of CAFs or using Sorafenib.

Furthermore, immunohistochemistry was applied to the ten FFPE samples of the aforementioned TMU LUAD patients. The results indicated that patients with low SVM_Score exhibit higher expression of alpha-SMA (Fig. 7a), which is used as a marker for activated myofibroblasts, and this trend is consistently observed in all ten patient tissues, with statistical significance (Fig. 7b). Similarly, the low SVM_Score patients demonstrate increased expression of COL5A1 (Fig. 7c), which is consistently significant across all ten patient tissues (Fig. 7d). Our study indicates that increased mechanical stress activates the EMT pathway (Fig. 1c, d), concurrently highlighting the reported association between the secretion of COL5A1 and tissue mechanical stress [32]. Therefore, multicolor immunofluorescence analysis was employed to investigate the relationship between COL5A1 and the tumor cell EMT pathway in the tissues of ten lung adenocarcinoma patients. The results revealed that low SVM_score tissues exhibited higher COL5A1 expression, and near the areas with elevated COL5A1 expression, Vimentin was also highly expressed (Fig. 7e). This trend is consistent across all ten patients and is statistically significant (Fig. 7f, g). Furthermore, in-depth statistical analysis of multicolor immunofluorescence shows that the number of COL5A1-positive cells is directly proportional to the number of vimentin-positive cells with a *p*-value less than 0.0001 (Fig. 7h).

Simultaneously, two myofibroblast strains, CAF1 and CAF2 (Supplementary Fig. 5a), were extracted from the LUAD tumor tissues. Small RNA interference downregulates COL5A1 in these two myofibroblast strains(Supplementary Fig. 5b, c). When co-cultured with tumor cells, it was observed that tumor cells located near CAFs with reduced COL5A1 expression also exhibited decreased Vimentin expression (Fig. 7i). We employed Transwell assays to investigate alterations in the invasive capacity of lung adenocarcinoma cells co-cultured with cancer-associated fibroblasts (CAFs). The co-cultivation of lung adenocarcinoma cells with CAFs led to an increase in the invasive ability of lung adenocarcinoma cells (Fig. 7j). However, when we knocked down COL5A1 in CAFs or treated them with Sorafenib, the invasive capacity of lung adenocarcinoma cells co-cultured with CAFs decreased compared with scrambled transfected CAFs (Fig. 7k).

### Sorafenib attenuates the tumor-promoting effect of COL5A1 from myofibroblast

**Fig. 8:**
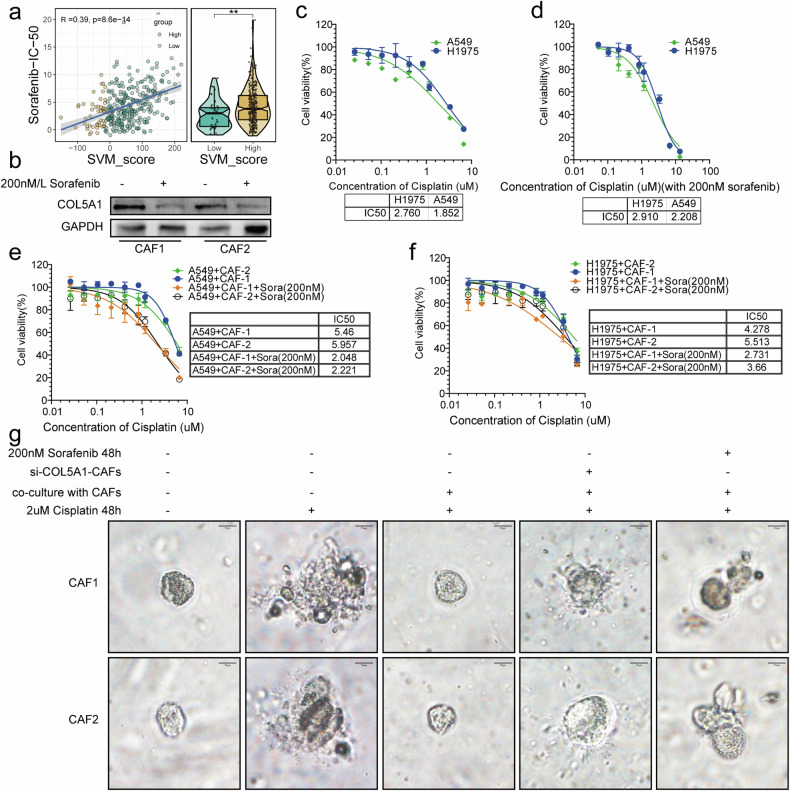
Sorafenib and knocking down of myofibroblast COL5A1 sensitize the lung cancer cells to cisplatin. **a** Correlation between Sorafenib IC-50 values and SVM_Score in TCGA cohort. **b** Demonstration of the impact of Sorafenib on COL5A1 expression in both CAF cell lines through Western blot. Determination of IC-50 values for cisplatin in A549 and H1975 cells (**c**), highlighting the impact of Sorafenib treatment on cisplatin IC-50 in H1975 cells (**d**). **e**, **f** IC-50 values for cisplatin after co-culture with CAF cell lines, with and without Sorafenib treatment, demonstrating the impact of Sorafenib on drug sensitivity. **g** Analysis of the response of organoids to cisplatin in co-culture with CAF cells and COL5A1-knockdown CAF cells, with and without Sorafenib treatment, to assess changes in drug sensitivity within the tumor microenvironment.

Previous studies have reported that Sorafenib decreases the expression of collagen and fibronectin genes, ultimately contributing to the reduction of tumor-stroma stiffness and concurrently alleviating intertumoral stress [21, 33]. To investigate whether Sorafenib attenuates COL5A1 from myofibroblasts, we first analyzed drug sensitivity in TCGA-LUAD, indicating a direct proportionality between the IC-50 and SVM_Score for Sorafenib, suggesting its potential use in treating patients with a poorer prognosis characterized by low SVM scores (Fig. 8a). Subsequently, we treated two myofibroblast cell lines with Sorafenib, revealing its inhibitory effect on COL5A1 expression (Fig. 8b). The IC-50 values for Sorafenib in these two myofibroblast cell lines were 4.133 µM/L and 3.955 µM/L, respectively (Supplementary Fig. 5d). Therefore, we selected a nonlethal concentration of 200 nM/L of Sorafenib for further treatment. CCK-8 experiments demonstrated that A549 and H1975 cells had IC-50 values of 2.76 µM/L and 1.852 µM/L for cisplatin, respectively (Fig. 8c). Posttreatment with 200 nM/L Sorafenib, there was minimal change in their IC-50 values for cisplatin (Fig. 8d).

Subsequent co-culturing of A549 and H1975 cells with the two CAF cell lines resulted in a nearly twofold increase in their IC-50 values for cisplatin (Fig. 8e, f). However, after treatment with 200 nM/L Sorafenib, the IC-50 values reverted to their original levels (Fig. 8e, f).

Concurrently, we established organoid models using patient-derived tissues from the same patients as the two CAF cell lines. When co-cultured with CAF cells, the organoids resisted cisplatin at 2 μM/L (Fig. 8g). Conversely, co-culture with COL5A1-knockdown CAF cells rendered the organoids vulnerable to 2 μM/L cisplatin (Fig. 8g). Similarly, treatment with 200 nM/L Sorafenib did not restore resistance to cisplatin when co-cultured with CAF cells (Fig. 8g).

## Discussion

The microenvironment of tumor tissues differs from that of normal tissues and is characterized by alterations in both biochemical and physical parameters [32]. Recent investigations underscore that, alongside biochemical cues, physical signals substantially influence cellular behaviors, such as proliferation and metastatic potential. Solid tumors exhibit distinct stiffening features, with tissue stiffness as an indicator for various human cancer types [34]. Tissue stiffness, as a biomechanical hallmark of solid tumors, has been the focus of extensive research and is believed to play a pivotal role in modulating diverse tumor characteristics, including growth, metabolism, invasion, metastasis, and therapy resistance [35, 36]. Meanwhile, as the major factors determining tissue stiffness, the quantity and cross-linking status of ECM components play crucial roles in malignant transformation and tumor progression. Notably, breast cancer tissue demonstrates a hardness approximately tenfold higher than that of normal breast tissue [37].

In contrast, the hardness of liver cancer tissue is analogously increased compared to normal liver tissue, owing to the association with chronic liver diseases that lead to hepatocellular carcinoma [38]. The activation of hepatic stellate cells in response to liver damage contributes to extensive ECM accumulation, thereby facilitating the progression of liver fibrosis, cirrhosis, and hepatocellular carcinoma [39]. Nevertheless, the precise mechanisms underlying how ECM stiffening propels tumor progression remain to be elucidated.

As demonstrated in our in vitro experiments, elevated mechanical stress remarkably enhances the migratory and invasive capabilities of LUAD cells. This underlines the multifaceted influence of mechanical cues on cancer cell behavior and characteristics, establishing mechanical stress as a pivotal player in tumor development.

To decipher the intricate interplay between genetic factors and the mechanical microenvironment, we introduced the SVM_Score. Constructed using Support Vector Machine analysis of basement membrane (BM)-related genes, this score emerged as a robust prognostic tool. The consistently high C-index across various datasets attests to its reliability and discriminative power. By validating various datasets, SVM_Score demonstrated robust prognostic prediction capabilities, with lower SVM_Score values corresponding to shorter overall and progression-free survival. SVM_Scores provide crucial prognostic insights and signify a paradigm shift in integrating genomic information with mechanical dynamics for personalized treatment strategies. A low SVM_Score also tends to have higher Ki67 positive and increased expression of Vimentin and indicates that SVM_Score is associated with tumor proliferation and EMT propensity in our in-house LUAD patients cohort. We also found that the tissues with different SVM_Scores have different mechanical properties, evidenced by using atomic force microscopy, and low SVM_Scores tend to have higher tissue stiffness. This demonstrated that SVM_Score is a factor that reflects the tissue mechanical characteristics and that the tissue mechanical properties are associated with prognoses and biological behavior, such as migration, invasion, and drug resistance of tumor cells.

By analyzing single-cell sequencing data from LUAD patients, we identified that myofibroblasts from the tumor tissues showed the lowest SVM_Score among all the cell populations. Our investigation revealed that collagen from myofibroblasts, with particular emphasis on COL5A1, plays crucial roles for SVM_Score. By analyzing the FFPE samples from LUAD patients, we found that LUAD tissues with Low SVM_Scores tend to have more myofibroblasts and COL5A1 and high Vimentin expression. Furthermore, in vitro experiment using siRNA knocking down the COL5A1 expression of myofibroblasts and co-culturing with lung adenocarcinoma cells, the Vimentin expression was downregulated, as well as the migration and invasion ability of lung adenocarcinoma cells.

Our research revealed the pivotal role of COL5A1 in influencing the mechanical properties of the tumor microenvironment. Our findings illuminate the intricate relationship between COL5A1, mechanical stress, and the activation of the Epithelial-Mesenchymal Transition (EMT) pathway, offering critical insights into LUAD progression. Both experiments on lung cancer patient tissues and in vitro experiments on lung cancer cells have demonstrated the role of COL5A1 in regulating the mechanical properties of the tumor microenvironment. Meanwhile, this is consistent with previously published research, where COL5A1 is closely related to the mechanical stress of tissues [40].

Our research found that by inhibiting COL5A1, Sorafenib-treated myofibroblasts inhibited adenocarcinoma cells’ invasion ability and sensitized adenocarcinoma cells to cisplatin. This inhibition of fibrosis makes Sorafenib a promising drug for regulating mechanical stress in tumors and inhibiting tumor malignancy. Therefore, our study provides a theoretical basis for applying Sorafenib to lung cancer.

The strong association between SVM_Scores and patient outcomes underscores the clinical relevance of our findings. Our results indicate that lower SVM_Scores correlate with shorter overall and progression-free survival, outperforming traditional clinical factors in certain datasets. Moreover, the lower immunogenicity observed in tumors with low SVM_Scores suggests a potential avenue for immunotherapeutic interventions.

Identifying COL5A1 as a key biomarker linking SVM_Scores and mechanical stress provides a foundation for further research and potential therapeutic targeting. The comprehensive analysis of cellular interactions within the tumor microenvironment, especially the prominence of myofibroblasts and their intricate communication pathways, opens new avenues for understanding the stromal dynamics in LUAD.

## Conclusions

Our study advances the understanding of the intricate interplay between mechanical stress, SVM_Scores, and collagen secretion from myofibroblasts in LUAD. Integrating innovative prognostic tools, mechanistic insights into tumor-stroma interactions, and identifying Sorafenib as a modulator of COL5A1 expression collectively contribute to the evolving landscape of precision oncology. This research lays the groundwork for future investigations to translate these findings into clinically relevant therapeutic strategies for patients with LUAD.

### Supplementary information

Supplementary figure

Supplementary Appendix 1

Supplementary Appendix 2

## Data Availability

The datasets [TCGA dataset] for this study can be found in the [cBioPortal] [http://www.cbioportal.org/]. The datasets [GSE3141 /GSE26939/GSE30219/GSE31210/GSE50081/GSE131907/GSE72094] for this study can be found in the [GEO Datasets] [https://www.ncbi.nlm.nih.gov]. The raw sequence data of TMU dataset reported in this paper have been deposited in the Genome Sequence Archive in National Genomics Data Center, China National Center for Bioinformation, Chinese Academy of Sciences (GSA-Human: HRA006222) that are publicly accessible at https://ngdc.cncb.ac.cn/gsa-human. The other data can be obtained from the corresponding author upon reasonable request.
