# Supplementary material for: Myofibroblasts derived type V collagen promoting tissue mechanical stress and facilitating metastasis and therapy resistance of lung adenocarcinoma cells
Source: Cell Death Dis. 2024 Jul 10;15(7):493. doi: 10.1038/s41419-024-06873-6 (PMC11237033; doi:10.1038/s41419-024-06873-6)
Supplement: Supplementary file 1 — Supplementary figure [file 41419_2024_6873_MOESM1_ESM.pdf]

Supplementary Figure:

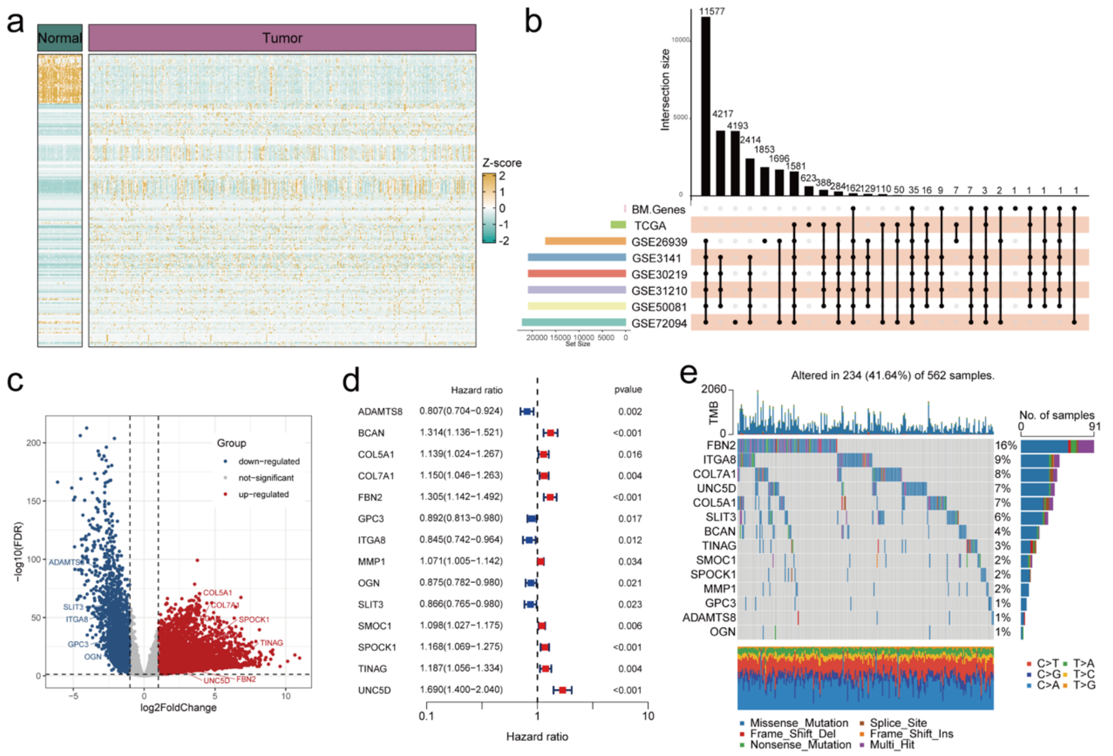

Supplementary Figure 1: Comprehensive analysis of BM genes and their relevance to lung adenocarcinoma. (a) A heatmap illustrating the comparison between normal and lung adenocarcinoma tissues from TCGA-LUAD provides an overview of the expression profiles of BM genes. (b) UpSet plot displaying the presence of 35 BM genes across all seven datasets used in the study, emphasizing the shared genes consistent in different datasets. (c) Volcano plot highlighting the differential gene expression between normal and lung adenocarcinoma tissues in the TCGA-LUAD dataset. (d) Univariate Cox regression analysis results reveal the identification of 14 BM genes related to the overall survival of patients with lung adenocarcinoma. (e) Oncoplot illustrating the mutation status of these 14 genes in lung adenocarcinoma.

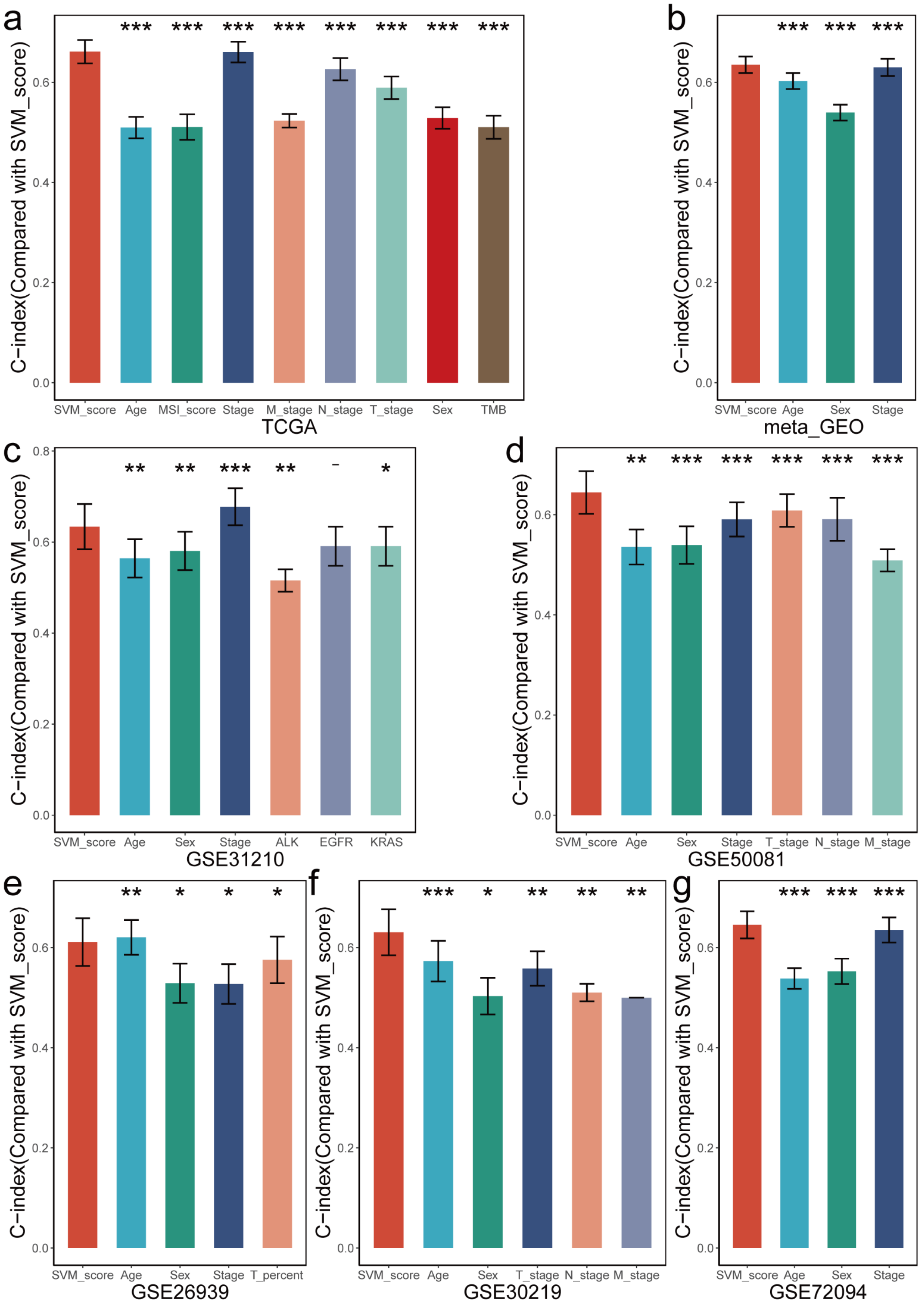

Supplementary Figure 2: Comparative analysis of SVM_Score with other commonly used prognostic indicators in various datasets. (a) Comparison of the c-index of SVM_Score with other clinical factors in the TCGA-LUAD dataset. (b) Comparison of the c-index of SVM_Score with other clinical factors in the meta-GEO dataset. (c-g)

Comparison of the c-index of SVM_Score with other clinical factors in all the GEO datasets.

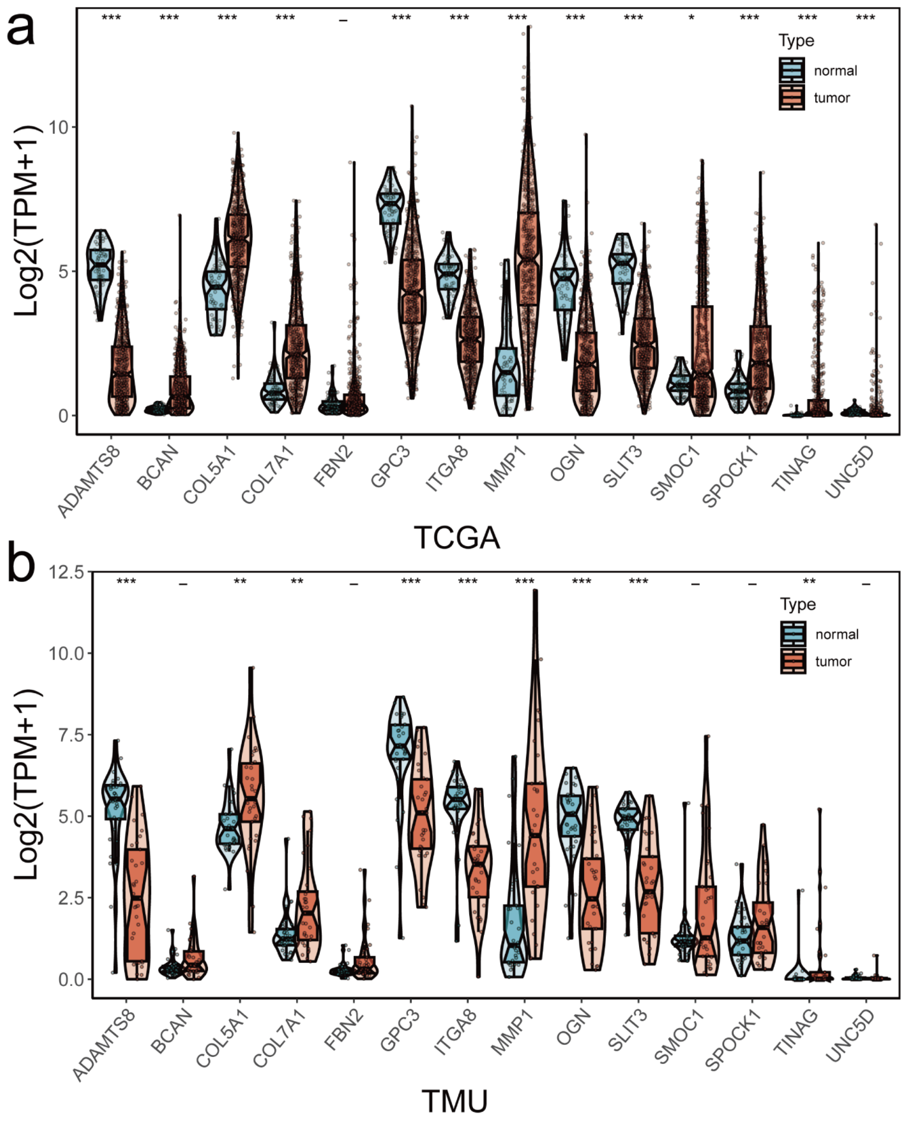

Supplementary Figure 3: Differential Expression of Genes Used for SVM_Score in Tumor and Adjacent Normal Tissues in TCGA and TMU Cohorts. (a–b) Analysis of the expression levels of genes used to construct SVM_Score in tumor and adjacent normal tissues in the TCGA-LUAD and TMU cohorts.

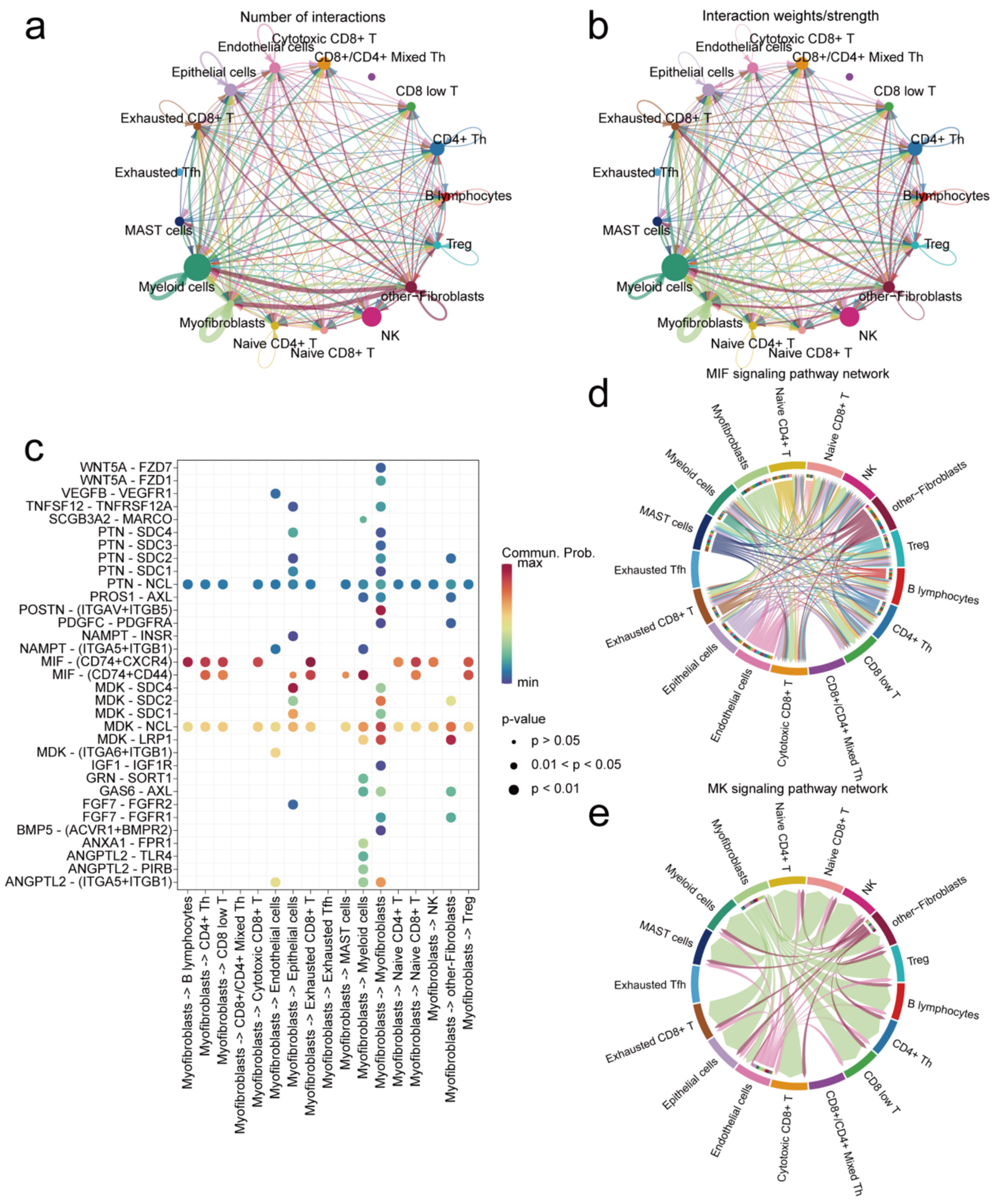

Supplementary Figure 4: Analysis of cellular interactions in the tumor microenvironment. (a) Evaluate the number of interactions between various cell types, emphasizing which exhibits the most significant interactions with other cells. (b) Assessment of interaction intensity among various cell types, highlighting which cell types exhibit the most meaningful interactions with others. (c) A dot plot was used to demonstrate the mode of intercellular communication (d-e). It focused on examining

interactions involving the MIF and MK signaling pathways, demonstrating their prominence in cell–cell communication within the tumor microenvironment.

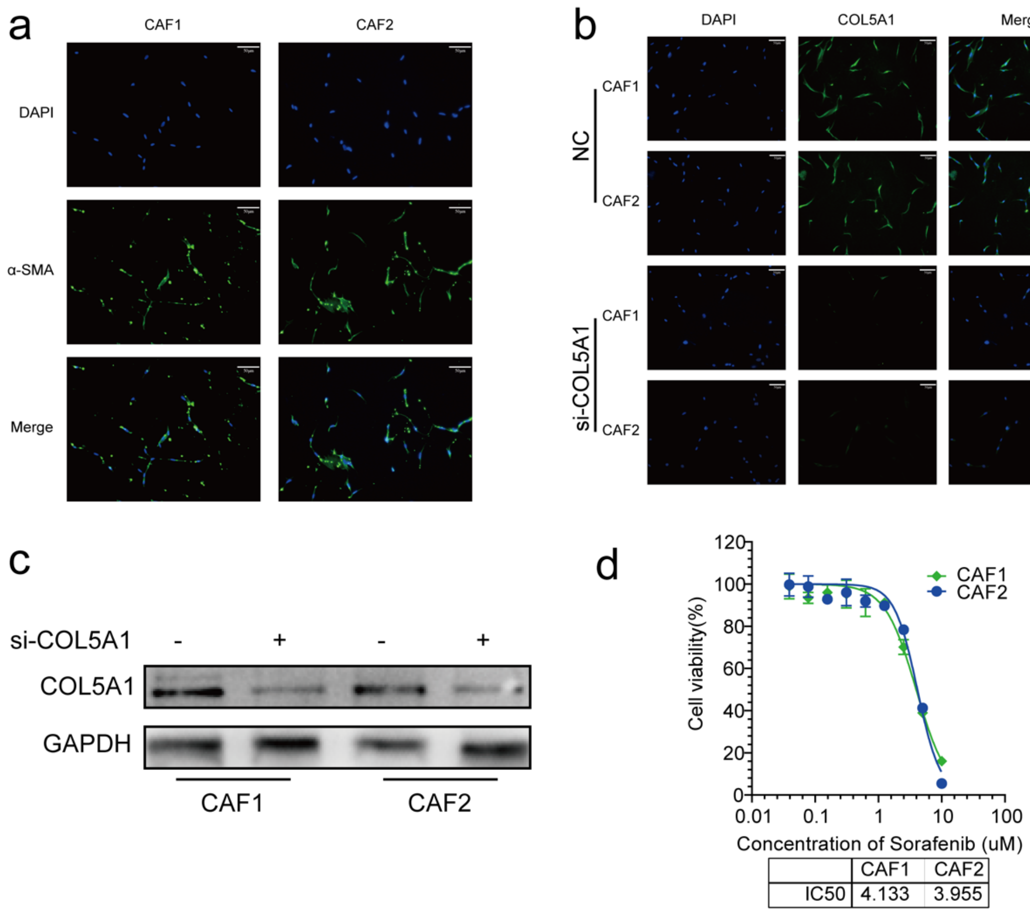

Supplementary Figure 5: Experimental procedures related to the identification of CAF cells and the experiments associated with COL5A1 and Sorafenib treatment. (a) Extraction of CAF1 and CAF2 from tumor tissues and their identification using alpha-SMA immunofluorescence. (b-c) Downregulation of COL5A1 in CAF1 and CAF2 cell lines using si-RNA interference, confirmed by immunofluorescence(b) and Western blot(c). (d) Determination of the IC-50 values of Sorafenib in both CAF cell lines.
